# Errors in Figures

**DOI:** 10.1001/jamanetworkopen.2026.33561

**Published:** 2026-08-18

In the Original Investigation titled “Enhanced Telehealth in Prostate Cancer,”^1^ published June 9, 2026, the unit of measure on the y-axes in Figure 1 and Figure 3B should be “No.,” not “%.” Some of the panel labels should include the following parenthetical information: “(35 total responses)” for Figure 1A and Figure 3B and “(25 total responses)” for Figure 1B. This article has been corrected.^1^
